# From the Field to the Emergency Room: A Systematic Case-Level Review of Exertional Rhabdomyolysis and Acute Kidney Injury in Athletes

**DOI:** 10.7759/cureus.111662

**Published:** 2026-06-28

**Authors:** Hajaj M AlHumaidan, Mohammed A Alrubaysh, Ahmad A Hakeem, Ahmad H Alsager, Alwaleed E AlSubaie, Malek K Albusair, Meshal A AlOhali

**Affiliations:** 1 Emergency Medicine, Prince Sultan Military Medical City, Riyadh, SAU; 2 Emergency Medicine, King Fahad Medical City, Riyadh, SAU; 3 Emergency Medicine, Independent Research, Riyadh, SAU; 4 Emergency Medicine, Brown University, Rhode Island, USA

**Keywords:** acute kidney injury, case review, dialysis, emergency medicine, exercise-induced muscle injury, exertional rhabdomyolysis, sports medicine

## Abstract

Exertional rhabdomyolysis is a potentially life-threatening syndrome caused by skeletal muscle breakdown with release of myoglobin and creatine kinase (CK) into the circulation. Clinical severity ranges from transient enzyme elevation to severe acute kidney injury (AKI) requiring renal replacement therapy (RRT). Although increasingly recognized, the published literature remains fragmented and largely descriptive. This review systematically aggregated case-level data to characterize biochemical severity and renal outcomes across different athlete populations and exertional contexts.

PubMed, Embase, Scopus, and Google Scholar were searched through December 2025 for published cases of exertional rhabdomyolysis with reported renal outcomes. Case-level data were extracted for demographics, athlete type, CK and creatinine values, author-defined AKI, RRT requirement, myoglobinuria, and recovery status.

46 articles met inclusion criteria, contributing 65 patients. Most patients were male (n=59, 90.8%), with a median age of 24 years (interquartile range (IQR) 20-30). The median peak CK was 55,000 U/L (IQR 25,000-120,000). AKI occurred in 49 patients (75.4%), and RRT was required in 14 patients (21.5%). Myoglobinuria was documented in 58 patients (89.2%). Recreational athletes represented the largest subgroup (n=20, 30.8%), followed by students or school athletes (n=14, 21.5%), military or law-enforcement personnel (n=12, 18.5%), collegiate athletes (n=7, 10.8%), manual laborers or occupationally active individuals (n=6, 9.2%), professional athletes (n=3, 4.6%), and other or unknown exertional contexts (n=3, 4.6%). No deaths were reported, and complete renal recovery was documented in 62 patients (95.4%).

Exertional rhabdomyolysis predominantly affects young, otherwise healthy men after unaccustomed or strenuous physical activity. Although AKI was common among published cases, survival and renal recovery were generally favorable. Standardized reporting of disease severity, AKI definitions, treatment strategies, and long-term renal outcomes is needed.

## Introduction and background

Rhabdomyolysis is a potentially life-threatening syndrome resulting from the breakdown of skeletal muscle fibers and the release of intracellular constituents-including myoglobin, creatine kinase (CK), potassium, and phosphate-into the circulation [1-3]. Clinically, it represents a final common pathway of diverse insults ranging from direct muscle trauma and strenuous exertion to metabolic, toxic, or infectious causes [2,4]. The global burden of rhabdomyolysis remains substantial, with approximately 26,000 cases reported annually in the United States and a wide spectrum of severity, spanning from asymptomatic enzyme elevation to severe acute kidney injury (AKI) and death [5].

The condition poses a significant diagnostic and therapeutic challenge within emergency and critical care medicine. Early recognition and prompt intervention are essential, as myoglobin-induced nephrotoxicity and electrolyte derangements can lead to substantial morbidity, including severe AKI and the need for renal replacement therapy (RRT) [1,6]. Despite decades of clinical recognition, rhabdomyolysis continues to result in preventable morbidity due to delayed diagnosis, inadequate fluid resuscitation, and underestimation of systemic complications [2,7].

From a pathophysiologic standpoint, skeletal-muscle injury induces sarcolemmal disruption and calcium influx, activating proteolytic enzymes and culminating in myocyte necrosis [3,8]. The liberated myoglobin, particularly in acidic or hypovolemic states, precipitates within renal tubules, obstructing urinary flow and generating oxidative injury to the kidney [6,8,9]. Consequently, AKI develops in up to 30-50% of hospitalized patients with severe rhabdomyolysis [9,10]. The diagnostic evaluation and clinical course of rhabdomyolysis have been well characterized in prior reviews emphasizing biochemical markers such as CK and myoglobin, which remain central to both diagnosis and prognostication [11-15].

Clinically, the syndrome spans multiple disciplines, including emergency medicine, nephrology, surgery, and sports medicine, and is increasingly reported in association with extreme physical exertion, heat stress, and polypharmacy in older populations [1,2,16]. The widespread use of statins and other myotoxic agents, combined with the growing popularity of high-intensity exercise programs, underscores the continued relevance of rhabdomyolysis as a modern health concern [6,7,17].

Rhabdomyolysis affects individuals across all age groups and clinical settings, with etiologies varying according to population and circumstance. The condition is estimated to account for up to 10% of all AKI cases in intensive care units worldwide [5]. Epidemiologic data suggest that traumatic and non-traumatic mechanisms contribute nearly equally, reflecting the spectrum of both traditional and contemporary risk factors [18].

Traumatic rhabdomyolysis commonly follows crush injury, burns, or prolonged immobilization, particularly among disaster or accident victims [5]. In contrast, non-traumatic causes encompass a wide range of triggers, including strenuous exertion, heat stroke, seizures, infections, metabolic abnormalities, and drug or toxin exposure [5,19]. Exercise-induced rhabdomyolysis has gained increasing prominence with the rise of high-intensity training and endurance sports, wherein unaccustomed activity, dehydration, or environmental stress may precipitate profound muscle injury [1,6-8].

Accordingly, we conducted a systematic, case-level review of exertional rhabdomyolysis to characterize its biochemical severity, renal complications, and outcomes across athlete types and exertional contexts, thereby bridging the gap between isolated case reports and broader epidemiologic understanding [20].

## Review

Study design

This study was designed as a descriptive, literature-based aggregation of published cases of exertional rhabdomyolysis in which renal outcomes were reported. It synthesized individual patient data extracted from case reports and small case series. The primary objective was to characterize the clinical features, biochemical severity, AKI occurrence, RRT requirement, and recovery outcomes across various athlete types and exertional contexts.

Data sources and search strategy

A comprehensive literature search was conducted to identify case reports and case series describing exertional rhabdomyolysis among athletes or physically active individuals with reported kidney-related outcomes.Electronic searches were performed in PubMed/MEDLINE, Embase, Scopus, and Google Scholar from database inception through December 2025. The search strategy combined relevant keywords and Medical Subject Headings (MeSH) terms, including “rhabdomyolysis,” “exertional rhabdomyolysis,” “exercise-induced,” “athlete,” “sports,” “acute kidney injury,” “renal failure,” and “dialysis.” Reference lists of prior reviews and eligible studies were screened manually to identify additional reports. Only full-text, peer-reviewed, English-language human studies were included.

Eligibility criteria

Studies were eligible for inclusion if they met all the following criteria: reported a case report or case series with individual-level data (each patient described separately). Described rhabdomyolysis primarily induced by physical exertion, sports participation, or occupational activity. Documented at least one biochemical indicator of rhabdomyolysis, typically a markedly elevated CK concentration. Reported at least one kidney-related outcome, such as serum creatinine level, AKI diagnosis, or dialysis/RRT. Provided sufficient clinical detail to allow extraction of demographic and laboratory variables.

Exclusion criteria

Exclusion criteria were as follows: rhabdomyolysis secondary to crush injury, major trauma, sepsis, toxins, drugs, infections, or metabolic disorders in which exertion was not the primary cause. Reports lacking individual-level data or usable renal outcome information. Non-human studies, conference abstracts without complete data, and non-English publications.

Study selection

**Figure 1:**
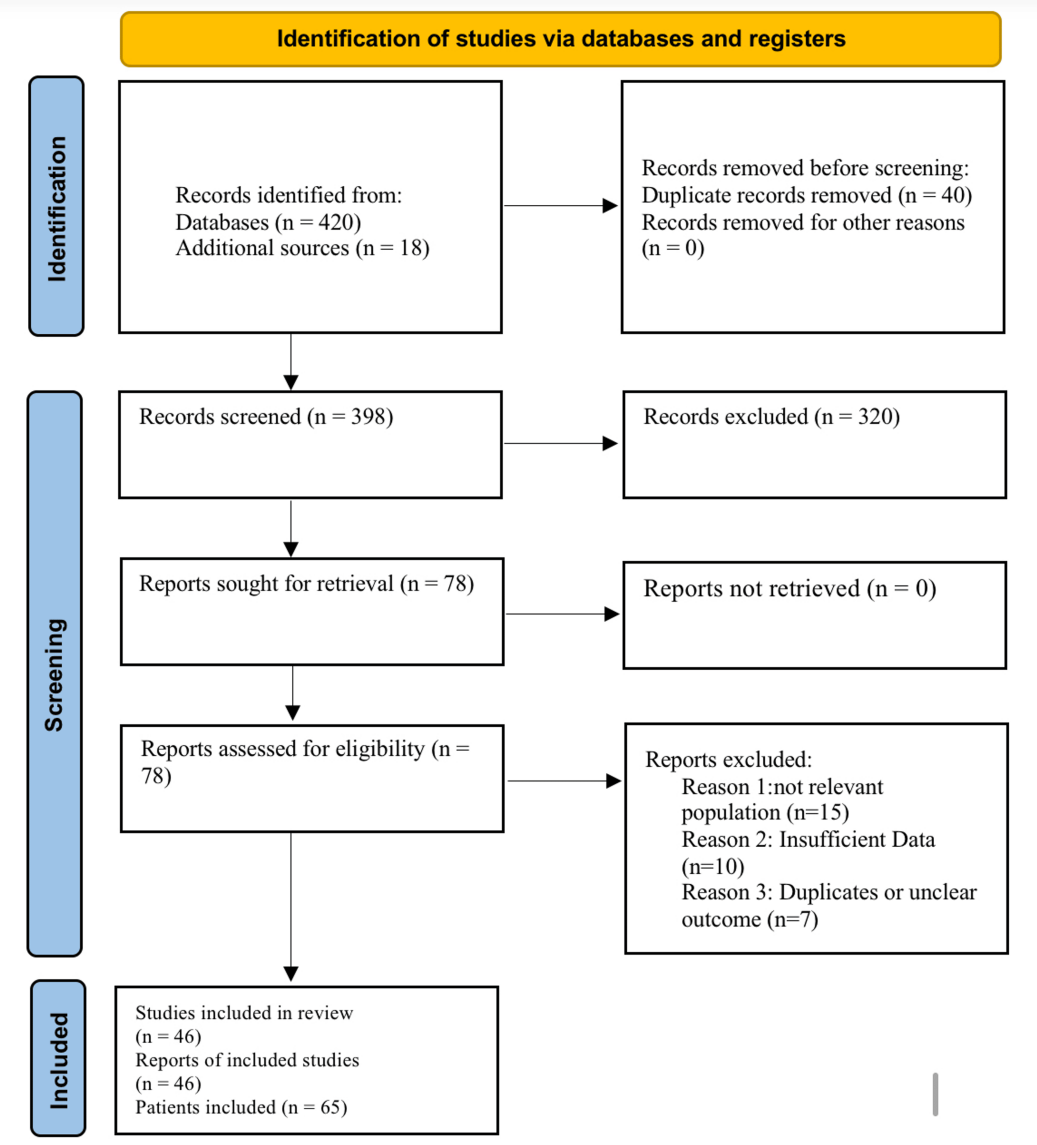
PRISMA flow diagram of study selection PRISMA: Preferred Reporting Items for Systematic Reviews and Meta-Analyses

After removal of duplicates, all titles and abstracts were screened for relevance to exertional rhabdomyolysis. Full-text articles were then assessed against the predefined eligibility criteria. A total of 46 publications met inclusion criteria, representing 65 individual patients with exertional rhabdomyolysis and extractable renal data. The study selection process is summarized in a Preferred Reporting Items for Systematic Reviews and Meta-Analyses (PRISMA) 2020 flow diagram (Figure 1), which outlines the number of records identified, screened, excluded (with reasons), and included in the final analysis. The included case reports and case series are listed in the reference section and are cited collectively in this article [21-66].

Data extraction

Data were extracted into a standardized spreadsheet for each patient using a predefined variable dictionary. The following information was collected: bibliographic information including file name, DOI, publication year, and country; demographics including age and sex; athlete and activity characteristics including self-reported athlete type, sport or activity, and the nature or intensity of exertion; and biochemical and renal parameters including peak CK level (U/L), initial and peak serum creatinine (mg/dL), myoglobinuria, and blood urea nitrogen (if reported). Kidney outcomes included AKI, as defined by the original authors, and RRT, including the modality and number of sessions when available. Clinical outcomes included survival status and recovery outcome (full recovery or residual impairment). When a variable was explicitly reported as not available or not measured, it was recorded as such rather than inferred. All entries were manually reviewed to ensure accuracy and internal consistency. Peak CK and serum creatinine values were defined as the highest documented measurements during hospitalization, as reported in each original case. When multiple values were available, the maximum was used to represent biochemical severity.

Operational definitions

Athlete type was standardized into eight categories: professional athlete; collegiate or organized-team athlete; military, police, or firefighter; recreational or gym trainee; student or school athlete; manual laborer or occupationally active individual; non-athlete or other exertion (e.g., heavy housework or punishment); and unknown or not reported. AKI was recorded as Yes or No based on the definitions provided by the original authors, such as rising creatinine, oliguria, or KDIGO-like criteria when specified. RRT requirement was recorded as Yes or No, and any form of RRT, including hemodialysis, hemofiltration, or continuous RRT (CRRT), was coded as Yes regardless of duration or number of sessions. Myoglobinuria was recorded as Yes if gross pigmenturia or a positive urine myoglobin test was documented, and No otherwise.

Statistical analysis

Given that all patients were drawn from published case reports and small case series, analyses were primarily descriptive and exploratory. Continuous variables, including age, CK, and serum creatinine, were summarized as median with interquartile range (IQR). Categorical variables, including sex, athlete type, AKI occurrence, RRT requirement, myoglobinuria, survival, and recovery outcome, were expressed as counts and percentages. Outcomes were summarized overall and stratified by athlete type. No formal inferential subgroup comparisons were performed because of the small sample size, heterogeneous reporting, and publication bias inherent to case reports and small case series. Analyses were conducted using IBM SPSS Statistics version 29.0 (IBM Corp., USA) and Microsoft Excel 365.

Ethical considerations

This review was based exclusively on data extracted from previously published case reports and case series. No new human subjects were involved, and all data were publicly available and fully de-identified. In accordance with institutional and international research ethics guidelines, formal approval from an ethics committee was not required for this secondary analysis.

Results

Study Characteristics

**Figure 2:**
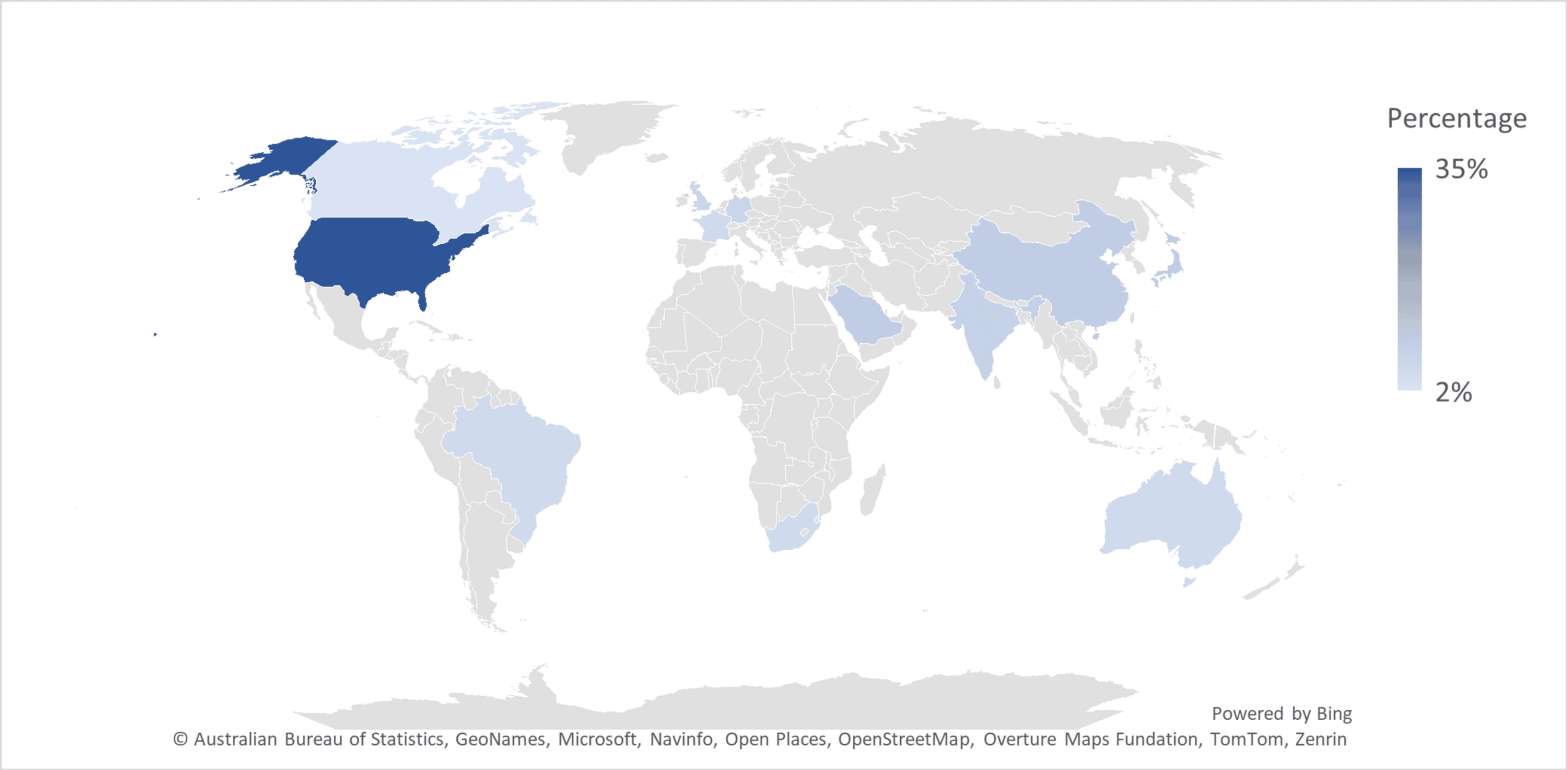
Geographic and temporal distribution of published exertional rhabdomyolysis reports with renal outcomes (2000–2025)

A total of 46 published articles met the eligibility criteria, contributing 65 individual patients with exertional rhabdomyolysis and reported renal outcomes. Most included publications were single-case reports (n=38, 82.6%), while the remainder were small case series (n=8, 17.4%) describing two to six patients each. Geographically, the majority of patients originated from the United States (n=31, 47.7%), followed by Asia (n=21, 32.3%), Europe (n=5, 7.7%), other regions in the Americas (n=5, 7.7%), and Africa or Oceania (n=3, 4.6%). Publication years ranged from 2000 to 2025 (Figure 2).

Demographics and Activity Context

**Figure 3:**
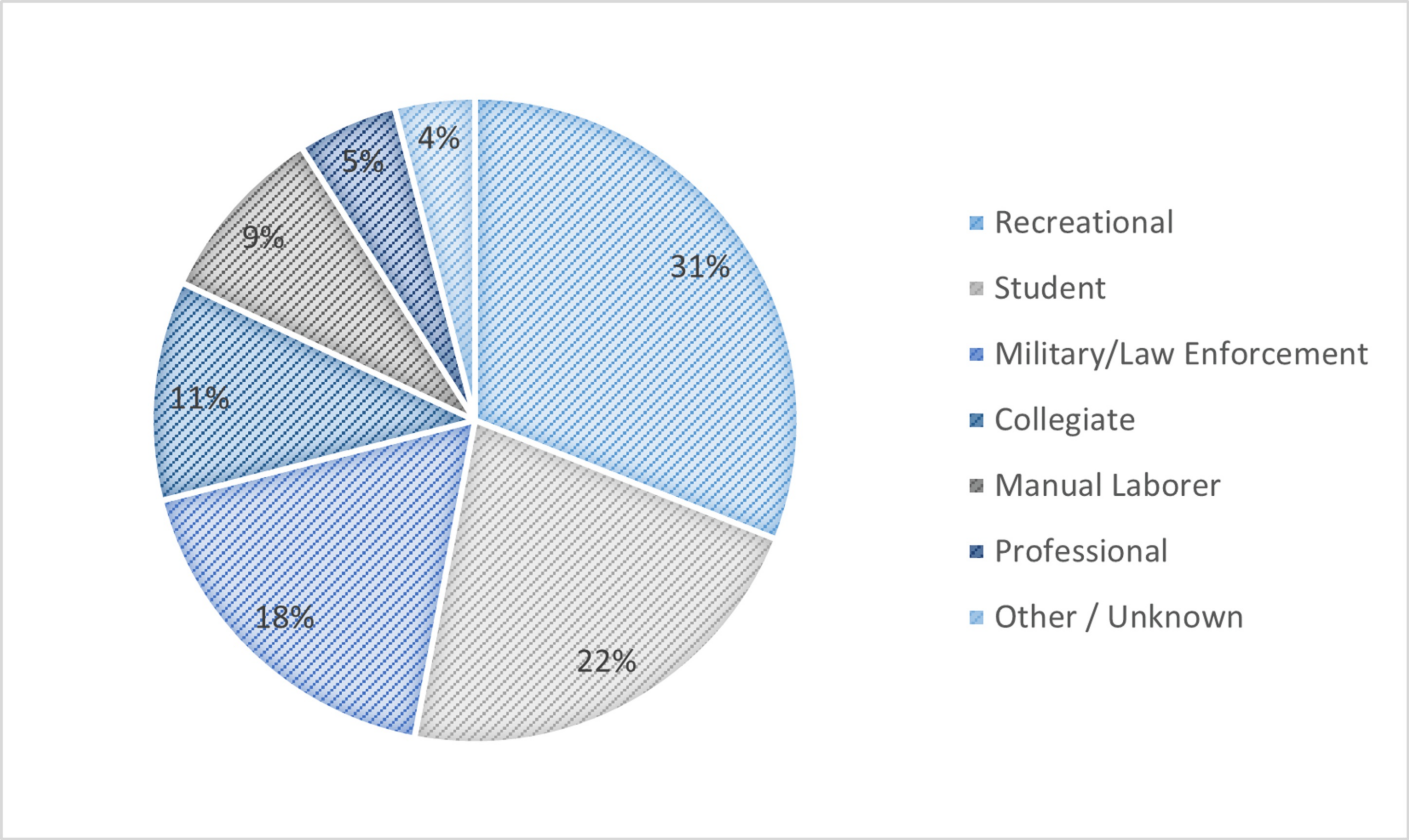
Distribution of exertional rhabdomyolysis cases by athlete type

Of the 65 included patients, most were male (n=59, 90.8%), and the median age was 24 years (IQR 20-30). All cases followed a clearly identified episode of strenuous or unaccustomed physical exertion, most commonly weight training, military drills, or team sports. When categorized by athlete type, the largest subgroup was recreational athletes (n=20, 30.8%), followed by students or school athletes (n=14, 21.5%), military or law-enforcement personnel (n=12, 18.5%), collegiate athletes (n=7, 10.8%), manual laborers or occupationally active individuals (n=6, 9.2%), professional athletes (n=3, 4.6%), and other or unknown exertional contexts (n=3, 4.6%) (Figure 3).

Biochemical Findings

**Figure 4:**
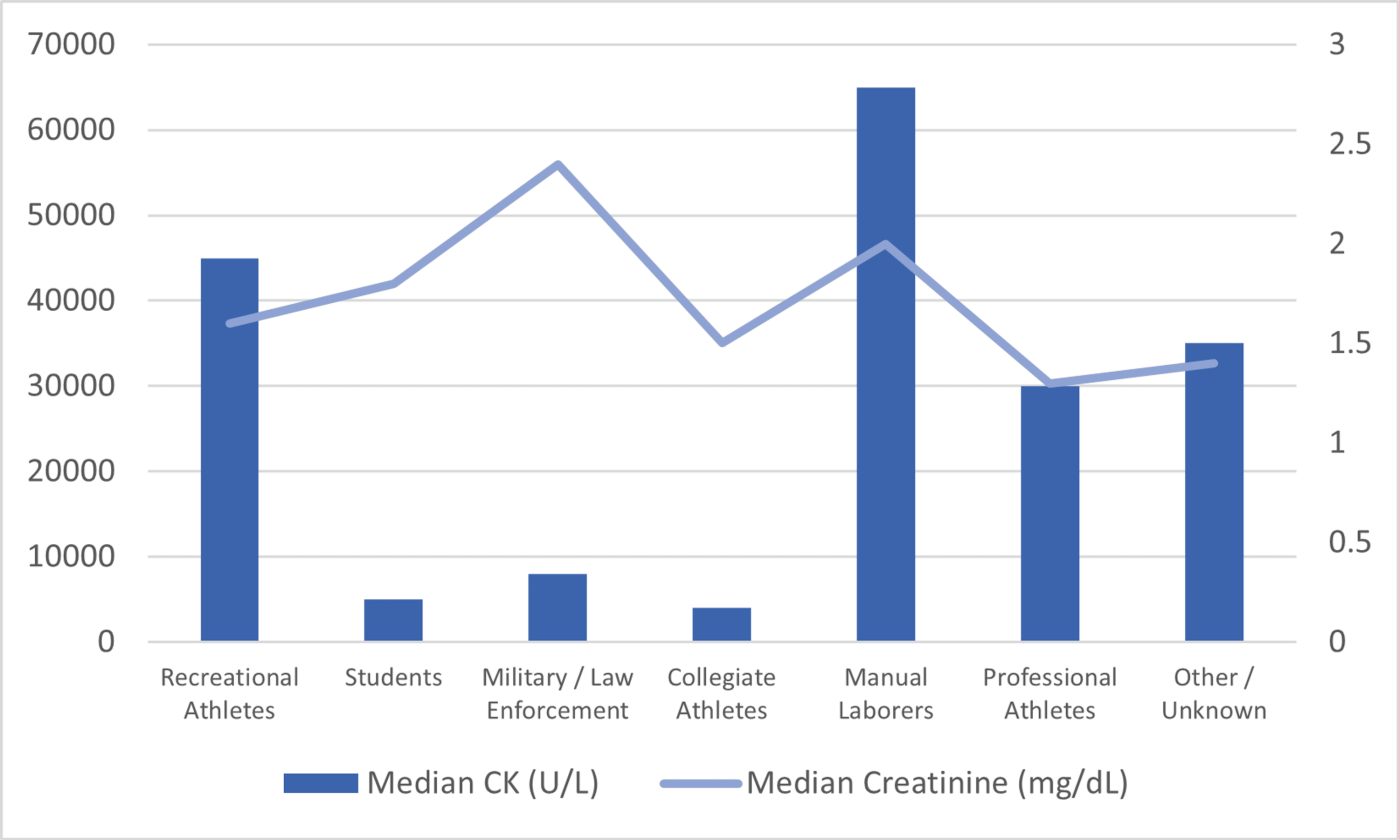
Peak CK and serum creatinine levels by athlete type CK: Creatine kinase

The median peak CK across all cases was 55,000 U/L (IQR 25,000-120,000). The median initial serum creatinine was 1.3 mg/dL, and the median peak serum creatinine among patients with AKI was 2.1 mg/dL. Myoglobinuria was documented in 58 patients (89.2%). Other electrolyte abnormalities, including hyperkalemia and hypocalcemia, were variably reported and were generally transient when described. Laboratory abnormalities typically normalized over a median of 7 days (range 3-21 days). Biochemical findings stratified by athlete type are shown in Figure 4.

Renal Outcomes

**Figure 5:**
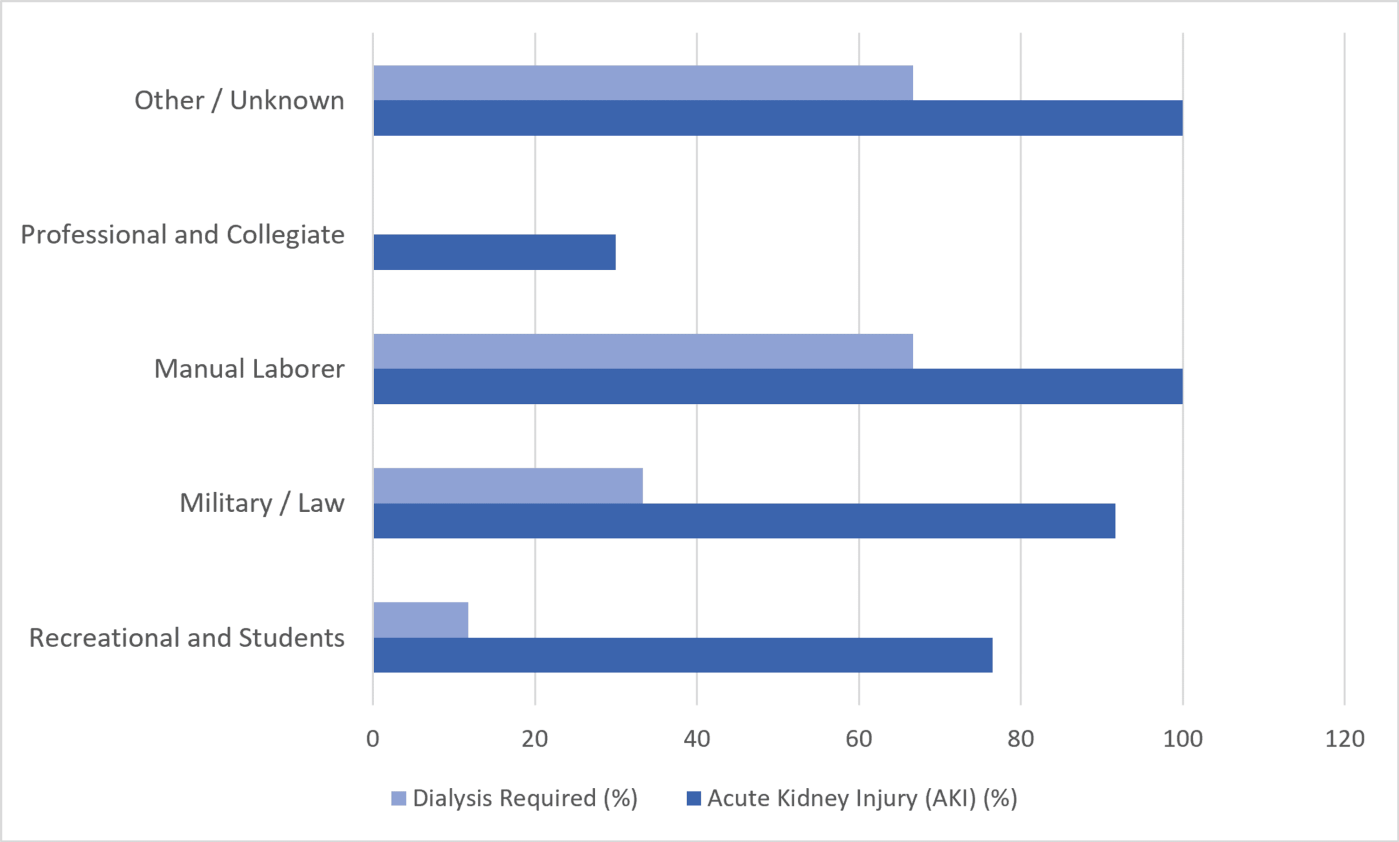
Renal outcomes by athlete type: AKI and RRT rates AKI: Acute kidney injury; RRT: Renal replacement therapy

AKI was reported in 49 of 65 patients (75.4%), and RRT was required in 14 of 65 patients (21.5%). All patients who received RRT survived and achieved complete renal recovery. The median time to renal recovery among RRT recipients was nine days, with a reported range of 5-20 days. When stratified by grouped athlete category, recreational and student athletes had AKI in 26 of 34 cases (76.5%), with RRT required in four of 34 cases (11.8%). Military or law-enforcement personnel had AKI in 11 of 12 cases (91.7%), with RRT required in four of 12 cases (33.3%). Manual laborers or occupationally active individuals had AKI in six of six cases (100.0%), with RRT required in four of six cases (66.7%). Professional and collegiate athletes had AKI in three of 10 cases (30.0%), and no RRT requirement was reported in this group, 0 of 10 cases (0.0%). Other or unknown exertional contexts had AKI in three of three cases (100.0%), with RRT required in two of three cases (66.7%) (Figure 5).

Clinical Outcomes

Overall survival was observed in all 65 patients (100%), and complete renal and functional recovery was achieved in 62 of 65 patients (95.4%) without long-term sequelae. A small number of patients reported transient fatigue or myalgia after discharge, but no chronic kidney dysfunction or recurrence of rhabdomyolysis was documented during follow-up.

Discussion

This review of 65 published cases represents one of the most detailed case-level analyses of exertional rhabdomyolysis and its renal outcomes to date. The findings delineate several clinically relevant patterns for emergency and sports medicine practitioners.

Demographic and Clinical Characteristics

Most affected individuals were young males, consistent with prior literature demonstrating a predominance among men engaged in high-intensity exercise or military training [12,18]. This observation likely reflects greater skeletal muscle mass, hormonal influences on exertional capacity, and male overrepresentation in physically demanding activities. The median peak CK concentration in this cohort exceeded 50,000 U/L - substantially above the diagnostic threshold of five times the upper limit of normal - confirming severe myocellular injury [1,3,12,16]. Despite markedly elevated enzyme levels, nearly all patients survived and achieved full recovery, emphasizing that early recognition and prompt fluid resuscitation can mitigate irreversible renal injury [1,4,17].

Renal Involvement and Dialysis

AKI developed in approximately three-quarters of cases, a proportion higher than the 30-50% typically reported in hospitalized rhabdomyolysis cohorts [4,10,17]. This likely reflects publication bias toward more severe or atypical presentations. RRT was required in about one-fifth of patients, and all patients who received RRT subsequently recovered renal function. These findings highlight the importance of early recognition, appropriate fluid resuscitation, correction of electrolyte abnormalities, and avoidance of nephrotoxic agents to limit the severity and duration of AKI and support renal recovery. RRT should be viewed as supportive therapy for severe AKI, refractory electrolyte abnormalities, or volume complications rather than as a direct determinant of long-term renal recovery [2,6,19].

Patterns by Athlete Type

When stratified by activity context, recreational athletes comprised the largest subgroup, followed by students and military personnel. Recreational exercisers most often developed rhabdomyolysis after unaccustomed or excessive training-frequently during “first-day” or return-to-gym sessions [1,6,8]. This pattern supports prior evidence that eccentric overload, dehydration, and insufficient recovery represent major precipitating factors among non-professional athletes [2,18].

Military and occupational cases appeared to have higher CK and creatinine values in this review. This may reflect prolonged exertion, heat exposure, delayed presentation, greater baseline muscle mass, or higher baseline creatinine in some individuals. Because baseline renal function, body composition, hydration status, and timing of presentation were inconsistently reported, these subgroup patterns should be interpreted cautiously [4,10]. In contrast, school and collegiate athletes appeared to have lower biochemical severity in the published cases, though this observation should also be interpreted cautiously because reporting and case-selection patterns varied across studies.

Myoglobinuria and Recovery

Nearly all cases reported myoglobinuria, reaffirming its diagnostic relevance [3,5,12]. Several authors noted, however, that urine discoloration resolved before CK concentrations reached their peak, underscoring the need for serial CK measurement even in the absence of visible pigmenturia [4,5,17]. In keeping with previous reports, almost all patients achieved complete clinical and renal recovery when treated promptly with intravenous fluids and vigilant electrolyte monitoring [2,6,19].

Comparison with Previous Literature

Earlier reviews of rhabdomyolysis have primarily encompassed mixed etiologies-traumatic, infectious, or drug-related-rather than exertional cases specifically [12,18]. Such studies often underreported key variables including temperature, hydration status, and exercise intensity. By aggregating individual-level data, the present analysis provides a more granular depiction of rhabdomyolysis occurring in otherwise healthy individuals.

The findings align with the pathophysiologic model described by Bosch et al. and Petejova and Martinek, wherein sarcolemmal disruption and intratubular myoglobin precipitation contribute to AKI [4,17]. They also reinforce prior recommendations advocating standardized reporting of CK kinetics, electrolyte profiles, and AKI staging to enhance comparability across studies [4,17].

Clinical and Research Implications

For emergency clinicians, these data underscore the importance of maintaining a high index of suspicion in patients presenting with myalgias, weakness, or dark urine following intense exertion. Serial CK and creatinine testing should be routine, even when initial results appear unremarkable. Preventive counseling regarding gradual training progression, adequate hydration, and scheduled rest remains essential for athletes and recreational gym participants [2,6,18].

Future investigations should focus on establishing prospective registries that capture standardized variables-such as exercise modality, environmental conditions, and management strategies-and evaluate novel early biomarkers, including neutrophil gelatinase-associated lipocalin (NGAL) and interleukin-6, for the prediction of AKI [12,17].

Limitations

This review has several limitations. Because the data were derived from published case reports and small case series, there is substantial potential for publication and selection bias, with more severe or atypical presentations more likely to be reported. Reporting was inconsistent for several clinically important variables, including ambient temperature, hydration status, fluid resuscitation protocols, timing of CK measurement, baseline renal function, and long-term follow-up. The possibility of duplicate reporting from multi-patient institutional series cannot be fully excluded. In addition, no formal risk-of-bias or quality assessment was performed because the included evidence consisted primarily of descriptive case reports and small case series. These limitations reduce the strength of inference, limit detailed subgroup analyses, and preclude causal conclusions.

## Conclusions

Exertional rhabdomyolysis most commonly occurs in otherwise healthy, physically active individuals following unaccustomed or intense physical exertion. In this case-level review, AKI was frequently reported, but survival and renal recovery were generally favorable among patients with available follow-up. These findings support the importance of early recognition, supportive management, and close monitoring of renal function and electrolyte abnormalities, while highlighting the need for standardized reporting of disease severity, treatment strategies, and long-term renal outcomes in future studies.
